# Cracks in Polymer Spherulites: Phenomenological Mechanisms in Correlation with Ring Bands

**DOI:** 10.3390/polym8090329

**Published:** 2016-09-02

**Authors:** Eamor M. Woo, Graecia Lugito

**Affiliations:** Department of Chemical Engineering, National Cheng Kung University, No. 1, University Road, Tainan 701-01, Taiwan; graecia_lugito@yahoo.com

**Keywords:** crack patterns, PLLA, ring-banded spherulites

## Abstract

This article reviews possible mechanisms of various crack forms and their likely correlations with interior crystal lamellae and discontinuous interfaces in spherulites. Complex yet periodically repetitive patterns of cracks in spherulites are beyond attributions via differences in thermal expansion coefficients, which would cause random and irregular cracks in the contract direction only. Cracks in brittle polymers such as poly(l-lactic acid) (PLLA), or poly(4-hydroxyl butyrate) (PHB), or more ductile polymers such as poly(trimethylene terephthalate) (PTT) are examined and illustrated, although for focus and demonstration, more discussions are spent on PLLA. The cracks can take many shapes that bear extremely striking similarity to the ring-band or lamellar patterns in the same spherulites. Crack patterns may differ significantly between the ring-banded and ringless spherulites, suggesting that the cracks may be partially shaped and governed by interfaces of lamellae and how the lamellar crystals assemble themselves in spherulites. Similarly, with some exceptions, most of the cracks patterns in PHB or PTT are also highly guided by the lamellar assembly in either ring-banded spherulites or ringless spherulites. Some exceptions of cracks in spherulites deviating from the apparent crystal birefringence patterns do exist; nevertheless, discontinuous interfaces in the initial lamellae neat the nuclei center might be hidden by top crystal over-layers of the spherulites, which might govern crack propagation.

## Preface

To sum up as a preface, the crack types in polymer spherulites may be grouped into three categories:

(A) Tri-branch star (or sometimes four-branch star) short cracks that usually occur in nuclei centers. Such crack pattern (tri-branch) is caused by parallel aligned sheaf-like lamellae orienting perpendicularly to substrates. The tri-branch cracks (cleaved usually when exposed to solvent or upon etching) are parallel to the shish crystal lamellae in nuclei centers.
(B) Radial short cracks that are parallel to the lamellae bundles radially-grown in spherulites. Such short radial cracks are indications of interfaces between the radially oriented lamellae in spherulites. These radial short cracks are usually segregated periodically by the circumferential cracks in the same spherulites.
(C) Circumferential long cracks that may assume four major patterns: (1) circular and concentric; (2) clockwise or counterclockwise spirals (similar to Archimedean spirals); (3) hexagonal shapes; or (4) hexagonal in inner portion, but circular in outer portion. Such long and circumferential cracks are often coinciding with the ring patterns commonly seen in spherulites.

Conventionally, cracks are dealt by thermal stress and contraction upon cooling from *T*_c_ to ambient (Δ*T*) owing to directional differences in thermal expansion (CTE) in the spherulites. Such models took account of only the directional differences in radial vs. tangential directions of spherulites, but did not look into the detailed lamellae assembly habits. As examples, PLLA spherulites have been found to exhibit as many as four crack types of different directions and patterns, which made it difficult to be explained simply by directional CTE. It should be emphasized here that differences in coefficients of thermal expansion certainly are present in any materials upon temperature changes; however, cracks of fixed patterns indicate that there might be other factors beyond the CTE differences. Mechanisms beyond the contraction via CTE differences are probed.

## 1. Introduction

Using gallstone spherulite crystals as models for covering all spherulites of organic compounds or polymers, Burns [1] illustrated relationships between stresses and cracks. Spherulites in general have spherical symmetry, but crystal properties are anisotropic in the radial and tangential directions. Cracks and ring-banded morphology in semicrystalline polymers have been extensively investigated and widely reported in the literature. The presence of large-size spherulites in a polymeric material can be the cause of brittleness, mainly via the formation of cracks that are expected to have negative impact on properties. Poly(l-lactic acid) (PLLA), like poly(4-hydroxy butyrate) (PHB), is a biodegradable polymer that is known to be brittle, possible owing to presence of cracks formed in cooling. Being brittle, they both are more prone to cracks-induced property deterioration. The optical sign of PLLA spherulites is of negative birefringence [2,3,4], while that of PHB is of positive birefringence [4,5]. However, both biodegradable polymers (PLLA and PHB) are capable of forming ring-banded spherulites when crystallized at suitable *T*_c_s, and developing various patterns of cracks upon cooling from *T*_c_s to ambient. PHB forms ring-banded spherulites more readily when melt-crystallized within a suitable range of *T*_c_s (usually *T*_c_ = 90 ± 20 °C). By contrast to PHB, PLLA is less distinct in showing ring-banded patterns in melt-crystallization. Formation of ring bands in PLLA can be influenced by some kinetic constraints, such as solution and melt vs. cold crystallization. In much earlier reports on neat PLLA, most investigators claimed that ring-band behavior could be observed only in solution-crystallized films, and not in melt-crystallized ones [3]. As examples, Sarasua et al. [6], and Gazano et al. [7] observed only ringless spherulites of PLLA from melt crystallization. Later, Xu et al. [8] demonstrated that ring-banded spherulites in neat PLLA could be obtained via cooling schemes, but isothermal melt-crystallization from the melt typically led to ringless spherulites. PLLA samples melted for longer time and PLLA grades with lower *M*_w_s and melting points are more likely to form ring-banded spherulites, as well as developing apparent cracks. The maximum temperature (*T*_max_) polymer melts are held before quenching for melt-crystallization at a *T*_c_ may also influence the spherulite patterns; this is to say that complete melting to erase the crystalline residues in PLLA may influence the tendency of formation of ring-banded spherulites. Moreover, Yasuniwa et al. [9] have reported that ring-banded spherulites in PLLA occur at *T*_c_ = 121–131 °C by melt-crystallization. Ring-banded PLLA spherulites may be seen when melt-crystallized at 125~140 °C. However, at same *T*_c_, ringless PLLA spherulites are resulted when the samples are subjected to cold-crystallization rather than melt-crystallization [10]. Apparently, behavior of ring bands in PLLA and many polymers had remained to be ellusive for a long time.

PHB has a chemical structure similar to PLLA; thus, it is informative to compare the spherulite morphologies of these two polymers especially in terms of banding and cracking behavior. Both PLLA and PHB crystallize into large-size spherulites in virgin forms. Behavior of cracks in crystallized PLLA or PHB has attracted attention of many investigators. In the case of PHB, two types of cracks have been reported: radial and circumferential cracks. Barham and Keller [5], who pioneered work on PHB cracks, have theorized that such circumferential cracks in PHB might have originated from the stress induced by the difference in thermal expansion coefficients (CTE) between the radial and circumferential directions during cooling process. Many investigators reported that crack formation in polymers occurs sometimes during crystallization, cooling processes, or by contacting with a solvent [11,12,13]. Other investigators, such as Martinez-Salazar et al. [14], have explained that the radial expansion coefficient is much greater than the circumferential one; thus, they theorized that a large tensile stress in the radial direction during the cooling process may be responsible. The result is circumferential cracks in spherulites. Nevertheless, an opposed question should be raised: lamellae in spherulites are grown and oriented in the radial direction with many lamellae being interfaced in the lateral direction; then, how can “radial” CTE be larger that circumferential CTE? That is, an alternative mechanism is necessary if this opposed question cannot feasibly answered.

In addition, complexity in cracks in polymers may be further puzzled by presence of glass substrates on samples. Hobbs et al. [15] have observed circumferential cracks during growth of PHB spherulites between two glass surfaces and found fine circles on PHB spherulites differing from the coarse shape of the cracks. On same PHB cracks, Xu et al. [16] proposed that those fine circles as “terraces” are resulted from instability of the moving boundaries of the confined molten film. Polymer samples crystallized with free top surface or confined in two glass slides may influence the cracks. Fraschini et al. [17] have discussed that circumferential cracks can also appear in ringless spherulites of PLLA in the constrained samples crystallized between glass slides only. As PLLA films are deposited on different substrates (glass, quartz, and Kapton), similar cracks are present upon cooling as long as polymer films are crystallized between two substrates. He et al. [18] have observed both circumferential and hexagonal cracks in ringless spherulites of PLLA melt-crystallized at 135 °C after quenching in liquid nitrogen, and they proposed that rhythmic growth and thermal shrinkage being the two main factors accounting for formation of periodic cracks.

In addition to neat polymers, cracks in polymer blends have also been reported. Recently, the crack behavior of PLLA in PHB/PLLA blend or racemic or non-equimolar PLLA/poly(d-lactic acid) (PDLA) complex systems [19] were investigated. For the PHB/PLLA binary crystalline/crystalline blend, Woo et al. [20] observed that radial cracks were found to aggregate on the circumferential bright bands (ridge band) but never on the dark bands (valley band) of spherulites, which are similar to those in neat PLLA. In the PHB/PLLA blend, crystallization takes place sequentially; the interlamellar structuring of PLLA at primary crystallization is superimposed by the PHB crystals at later stage, resulting in cracks patterns roughly governed by the lamellar assembly of PLLA (primary crystallization). These radial cracks, not circumferentially aligned, cannot be adequately interpreted by the temperature-induced coefficients of thermal expansion (CTE) upon cooling, as proposed earlier by Keller et al. [14] or Hobbs et al. [15]. In the non-equimolar PLLA/PDLA complex system, unmelted sc-PLA crystals serve as nuclei for later-crystallized PLLA [19].

Cracks in crystallized spherulites, like ring bands in poly(l-lactic acid) (PLLA), PHB, poly(trimethylene terephthalate) (PTT), and many other polymers have initiated objectives for this paper. Although cracks in polymers had long been attributed to stress differences between the circumferential and radial contractions are responsible; yet, many other factors may be at work beyond simple thermal contraction differences in two directions. Contraction owing to CTE differences in the radial vs. transverse directions may apply to explain the presence of smooth circular circumferential cracks in the ringless PLLA spherulites. However, CTE alone is not sufficient to explain the simultaneous presence of short radial cracks and dual circular cracks in the ring-banded spherulites. Mechanisms of cracks in polymer spherulites including PLLA have to be modified and updated, by realizing that there exist many potentially discontinuous interfaces between lamellae or their bundles in the radial and circumferential directions within the spherulites. This article will further expound about the possible sites of discontinuity and interfaces in the crystals of a ring-banded or ringless spherulite, and how these interfaces may act as weak spots of separation during cooling-induced contraction.

Typically, the spherulites in crystallized polymers show cracks upon cooling; yet in many circumstances, the ring-band morphology, and crystal lamellae therein, of the PLLA or PHB spherulites could be parallel to the crack patterns. This fact indicates that crystal lamellae assembly in spherulites, interfaces between the lamellae bundles, and their orientation patterns at specific *T*_c_ can further determine the formation and patterns of cracks upon cooling from *T*_c_ to ambient. For wider coverage, another ring-banded and crack-prone polymer, poly(trimethylene terephthalate) (PTT), is also discussed. The complexity between optical ring-band patterns and mechanical cracks is yet to be investigated in details, and rarely any reports have been dedicated to the issue of connection between the two critical phenomena in crystallized spherulites. Correlations between the cracks and ring-band morphology in spherulites of PLLA or other polymers were attempted in this study. This article aimed at interpretations based on phenomenological mechanisms of cracks in polymer spherulites that might have intimate correlation with ring bands.

Some reports in the classical literature tended to view the lamellae crystals in ring-banded spherulites as continuous spiral twist single-crystals orderly and synchronously packed to form optically birefringent patterns as elaborated in a review article by Lotz and Cheng [21], who continued and extended the original models much earlier proposed by Keller [22], and Keith and Padden [23,24], respectively. Such views of continuous spiral-twist lamellae, however, are not essential and perhaps become less practical based on several recent solid evidence from studies taking alternative approaches and with aids of recent advancement in instrument [25,26,27,28]. The intricate novel views on lamellae assembly in ring bands could shed new lights helping to understand the cracks in spherulites. By shedding new lights on such possible correlations, mechanisms on cracks can be further clarified.

## 2. Cracks in Ring-Banded vs. Ringless PLLA Spherulites

Cracks in constrained (free top surface) vs. unconstrained samples (sandwiched between two substrates) of LMw-PLLA (*M*_w_ = 11k) can be significantly different in ring bands, which correspondingly alter the cracks in spherulites upon cooling [29]. Using free surface vs. top confinement on PLLA films during crystallization, this example demonstrates that crack patterns vary with respect to ring bands. PLLA films upon crystallization with top confinement develop ring bands. Cracks also vary with respect to these two situations. Unconstrained PLLA samples upon crystallization do not show ring-banded spherulites; yet, they can show cracks upon cooling. Nevertheless, the cracks in ringless PLLA spherulites (free surfaces) differ completely with those in ring-banded PLLA spherulites (top-covered films). These facts suggest that as the lamellae and their assembly differ in spherulites (ringless vs. ring-banded), cracks may differ correspondingly.

**Figure 1:**
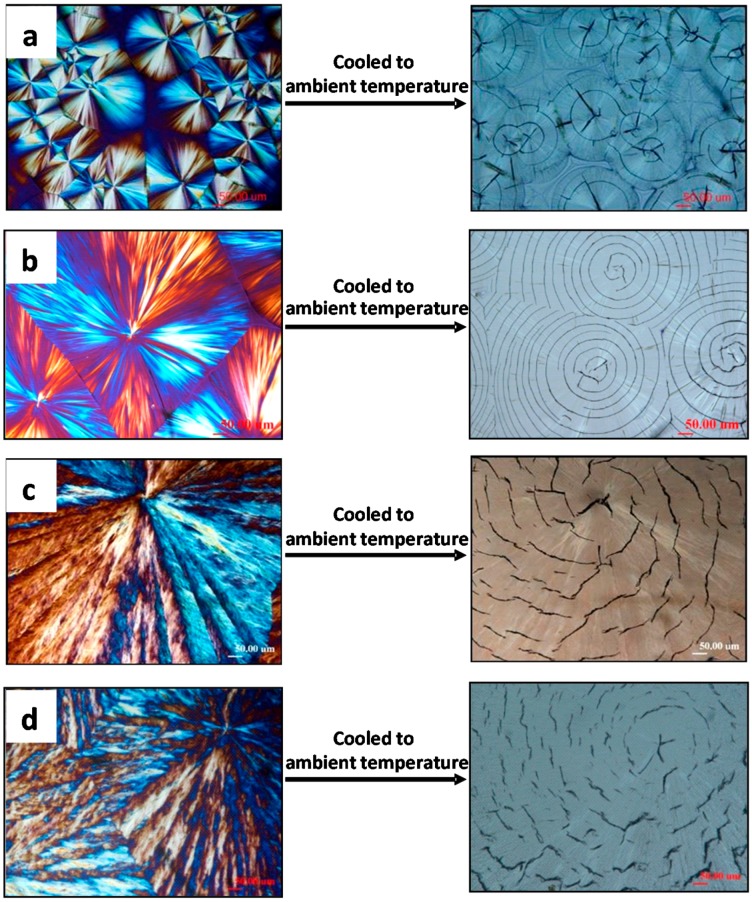
Cracks upon cooling from *T*_c_ as revealed in optical microscopy morphology of PLLA spherulites crystallized with no top cover (unconstrained) at *T*_c_: (**a**) 120 °C; (**b**) 125 °C; (**c**) 130 °C; and (**d**) 135 °C. Reproduced from Ref. [29] with permission from Springer (scale bar = 50 μm in all graphs).

Figure 1 shows that all PLLA (*M*_w_ = 11k) spherulites are ringless (film on glass substrate, uncovered) when crystallized at *T*_c_ = (Figure 1a) 120, (Figure 1b) 125, (Figure 1c) 130, and (Figure 1d) 135 °C, respectively, and cooled to ambient temperature. Note that the tri-branch star-like cracks at nuclei center are universally present in all PLLA samples crystallized at low or high *T*_c_s. O’Donohue, et al. [30] reported that even in natural gallstone crystals (taken from clinical gallbladders), there are tri-branch cracks (nick-named as “Mercedes-Benz sign” by authors), which are similar to those in the crystallized PLLA spherulites shown in this figure. Figure 1 further shows that circumferential cracks in PLLA(11k) spherulites apparently are in the uncovered/unconstrained samples (free top surfaces). One can see clearly that the cracks in the ringless PLLA spherulites change in a systematic trend (circular concentric to irregular circumferential, then finally to truncated zigzag arcs) as T_c_ is increased from low to high (120 to 135 °C). The lamellae in high *T*_c_-crystallized (135 °C) are apparently much coarser than those crystallized at lower *T*_c_, and they tend to have discontinuity between the lamellae in the radial direction, leading to discontinuous interfaces prone to be ruptured into visible cracks upon cooling-induced contraction. An earlier report by Fraschini et al. [17] claimed that circumferential cracks appeared only in ringless spherulites of PLLA that are crystallized in unconstrained samples. Our updated studies [29,31,32] have shown that, as a matter of fact, PLLA spherulites of ringless lamellar patterns could display circumferential crack types, regardless of constraints or not.

**Figure 2:**
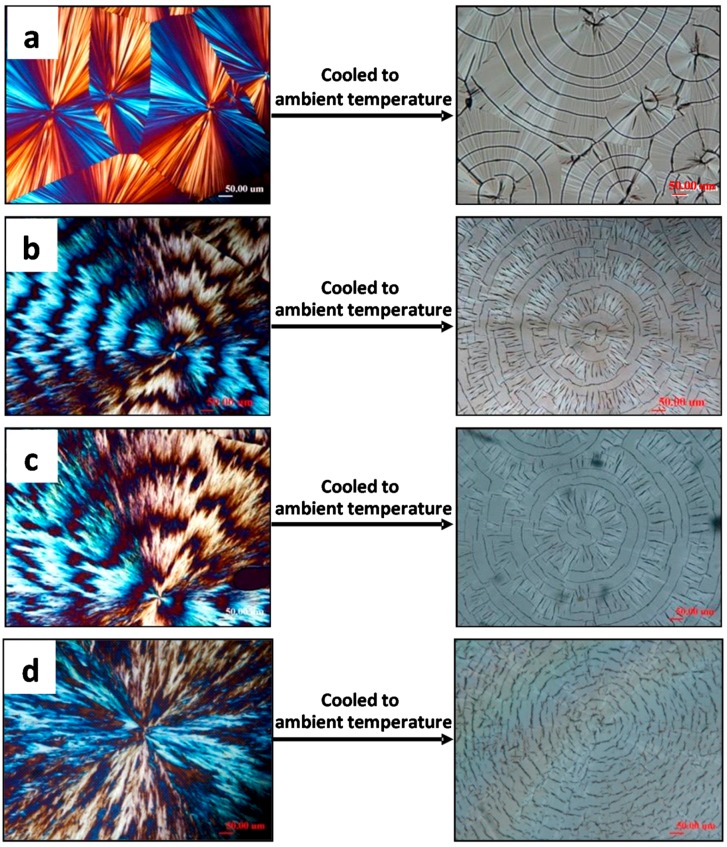
Cracks upon cooling from *T*_c_ as revealed in optical microscopy morphology of PLLA spherulites crystallized with a top cover at *T*_c_: (**a**) 120 °C; (**b**) 125 °C; (**c**) 130 °C; and (**d**) 135 °C. Reproduced from Ref. [29] with permission from Springer (scale bar = 50 μm in all graphs).

Top-covered PLLA films display entirely different patterns of cracks. Figure 2 shows POM and OM morphology of PLLA(11k) films crystallized with a top cover at *T*_c_ ranging from 120 to 135 °C, where distinct ring-banded spherulites appear at *T*_c_ = 125 and 130 °C, respectively. For the samples crystallized at 120 °C or 135 °C, the PLLA spherulites are ringless with smooth lamellae of fine and thin textures. Note that for these two ringless PLLA spherulites (*T*_c_ = 120 and 135 °C, respectively), the cracks are correspondingly similar to those seen in the ringless PLLA spherulites discussed in Figure 1. However, for samples crystallized at 125 and 130 °C, the PLLA spherulites show distinct ring bands with alternating bright and dark bands of the rings, with the dark bands apparently owing to light extinction. These ring-banded PLLA samples crystallized at *T*_c_ = 125 and 130 °C, upon being cooled to ambient, develop cracks (short radial cracks + twin circumferential cracks) that are dramatically different from those in the ringless PLLA spherulites crystallized at same *T*_c_ = 125 and 130 °C shown in Figure 1. Note carefully that there are actually “dual-rings”, whose positions roughly coincide with the optical dark-bright transitions of alternating bands in the ring-banded spherulites. More precisely, the twin circumferential cracks are located and coincide with the boundaries of the bright and dark bands. For PLLA films crystallized at any *T_c_* with or without top confinement, the lamellae there in the ring-banded spherulites or ringless ones are intimately correlated to the crack types formed during cooling from *T*_c_ to ambient.

Cracks and their formation in spherulites may be influenced by the crystallinity or *M*_w_s, and, thus, brittleness of polymers. Using PLLA grades of three different *M*_w_s (11k, 120k, and 258k) as examples, Woo et al. [31] have shown that multiple types of cracks, i.e., circumferential and/or short radial cracks, are massively present in low-molecular-weight PLLA (LM_w_-PLLA-11k), which has a high crystallinity and probably the least mechanical strength. Upon cooling the LM_w_-PLLA-11k from most crystallization temperatures, cracks easily form and the crack patterns are dependent on the lamellar morphology within the spherulites as already discussed in Figure 1 and Figure 2. As proven by Woo et al. [31], the crystallinity of PLLA of different grades varies from 73% (measured by DSC) for LM_w_-PLLA-11k to 47% for HM_w_-PLLA-258k. With a higher M_w_-PLLA-120k, hexagonal cracks, rather than circular circumferential ones, occur spontaneously during cooling process to ambient after crystallized at high *T*_c_ = 135 °C. The crack pattern of PLLA-120k films (either with or without a top cover) crystallized at 135 °C is of a hexagonal geometry. Although it is intuitive to attribute this hexagonal pattern of cracks to hexagonal (110) growth plane, as He et al. did [18]; nevertheless, it is difficult to rationale why at other *T*_c_s or for other PLLA grades, the cracks in spherulites are circular, instead of hexagonal. We will further expound the hexagonal-shape cracks later by correlating with hexagonal ring bands in PLLA spherulites. For even higher M_w_-PLLA (HM_w_-PLLA-258k), no cracks of any types (either circumferential or radial cracks) are present in PLLA films crystallized at *T*_c_ = 135 °C upon either slow cooling or quench-cooling by dipping the samples into liquid nitrogen. The high-M_w_ PLLA usually has a lower crystallinity and higher mechanical properties and less brittleness. It should be noted that the HM_w_-PLLA-258k crystallized at *T*_c_ = 130–135 °C does not develop the extinction–band rings as seen in LM_w_ PLLA grades (11k or 120k); thus, as a result, it does not show cracks as those seen in LM_w_ PLLA upon cooling (except for a few inter-spherulite boundary voids). These observations demonstrate yet another strong correlation between lamellar assembly in spherulites and crack patterns, indicating most of the cracks (though not all types) tend to follow the assembly pattern of lamellae in spherulites (either ring-banded or ringless) and detachment into cracks most likely occurs at interfaces of discontinuity among lamellae.

**Figure 3:**
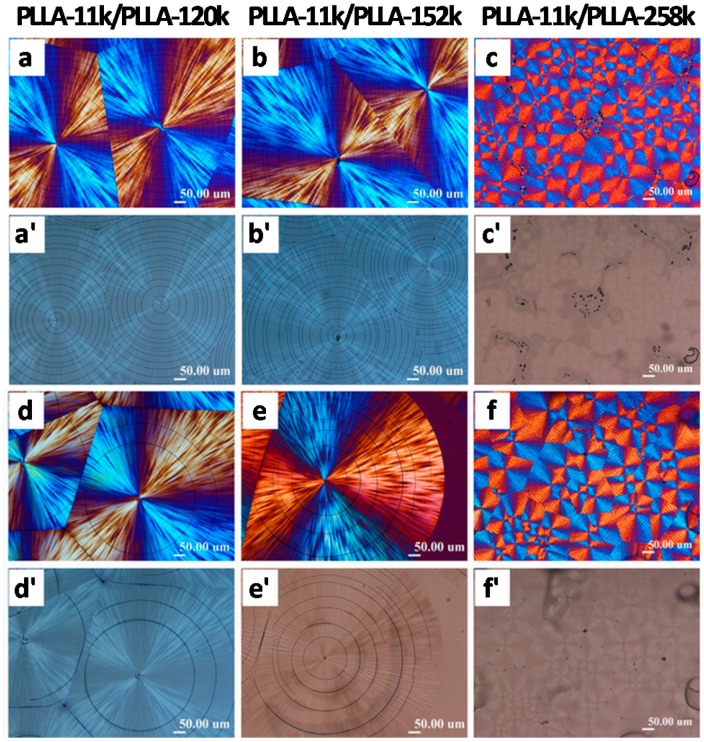
POM (a–f) and OM (a’–f’) micrographs showing cracks and corresponding spherulitic morphologies of LM_w_-PLLA blended with HM_w_ PLLA (50/50) melt-crystallized at *T*_c_ = 130 °C and cooled to ambient: (**a**–**c**) samples with top cover (constrained); and (**d**–**f**) samples without top cover. Reproduced with permission from Ref. [31].

Interestingly, as LM_w_-PLLA (11k) is blended with HM_w_-PLLA (152k or 258k) at 1:1 ratio, crystallized at same *T*_c_ = 130–135 °C and cooled, the crack patterns are entirely different from those in neat LM_w_-PLLA (11k) or neat HM_w_-PLLA (152k or 258k). The LM_w_-PLLA/HM_w_-PLLA (1:1) blend displays an entirely different spherulitic morphology and the crack patterns of the LM_w_/HM_w_ blend also follow the lamellae assembly. Figure 3 shows cracks and spherulitic birefringence patterns in PLLA-11k blended (1:1) with PLLA-152k and PLLA-258k, respectively, at *T*_c_ = 130 °C and cooled to ambient. Both uncovered samples (Figure 3a) and uncovered samples (Figure 3b) are shown for comparison. For either covered or uncovered LM_w_-PLLA/HM_w_-PLLA blend samples, the spherulitic birefringence and cracks are similar, except that the crack density are higher in the covered blends (i.e., inter-crack spacing is smaller) than the uncovered samples. Apparently, the spherulitic birefringence of the LM_w_/HM_w_ blend (50:50) resembles neither that of neat LM_w_-PLLA-11k nor that of neat HM_w_-PLLA (152k or 258k), and so do the crack patterns. Once again, the fact further reinforces that as the spherulite birefringence is changed (reflecting variation in lamellae assembly in spherulites), the crack pattern will correspondingly vary. For neat LM_w_-PLLA (11k), there are distinct extinction rings in the birefringent spherulites, with twin circumferential cracks superimposed on the extinction rings, and short radial cracks truncated by the circumferential cracks (as shown earlier in Figure 1). For the LM_w_-PLLA-11k/HM_w_-PLLA-152 or LM_w_-PLLA-11k/HM_w_-PLLA-258k blends, neither extinction rings nor twin circumferential or short radial cracks are present.

LM_w_-PLLA (11k) crystallizes into ringless or ring-banded spherulites depending on *T*_c_, *M*_w_, and/or cover confinement, leading to two categories of the crack patterns. In the ring-banded LM_w_-PLLA spherulites, two different crack types are present: (I) twin-circumferential cracks in the transitional boundaries; and (II) radial short-segmental voids locating on the bright (ridge) bands in spherulites. If the high-M_w_ PLLA is blended with LM_w_-PLLA, crack forms in the blends are dramatically modified, owing to the fact that the ring band behavior in the blends is dramatically different from those in the LM_w_-PLLA. For the LM_w_-PLLA-11k/HM_w_-PLLA-152k or LM_w_-PLLA-11k/HM_w_-PLLA-258k blends, neither extinction rings nor twin circumferential or short radial cracks (observed in neat LM_w_-PLLA) are present, but the crack forms in the LM_w_-PLLA/HM_w_-PLLA blends more resemble those in the neat HM_w_-PLLA.

## 3. Temperature-Dependence and Reversibility of Cracks

Consistently, when viewed in an optical microscopy, cracks are not observable in crystallized polymer samples when held at *T*_c_ without cooling; nevertheless, ring bands or crystal orientations in spherulites are visible at this *T*_c_. When the *T*_c_-crystallized polymer samples are cooled down to ambient (or any temperatures much lower than *T*_c_), cracks of various types appear upon cooling, whose patterns depend on the ring-banded or ringless morphologies. Apparently, crystallization temperature, or other kinetic factors (diluents, solvents, confinement by substrates, etc.), constitutes some of the main factors affecting the spherulite morphology, which in turn determines how the crack patterns may appear in final cooled spherulites. Although smooth single circumferential cracks in PLLA ringless spherulites had been widely disclosed in the literature, the simultaneous presence of twin-circumferential and short-radial cracks in the ring-banded LM_w_-PLLA-11k spherulites were first reported by Woo et al. [29]. Apparently, for the same material, the cracks in the ringless and ring-banded PLLA spherulites, both crystallized at same *T*_c_ but with free surface vs. top confinement, respectively, differ significantly. Given the same contraction owing to CTE, the fact of dramatically different crack patterns suggests that ring-banded spherulites have different lamellae assembly from the ringless one, leading to different cracks. A recent study [29] has revealed that the cracks would be reversible with temperature heating/cooling cycles; that is, cracks in cooled samples would disappear if the samples are brought from ambient to *T*_c_ or above, and then would re-appear again upon cooling. For the cracks of all three or four types in crystallized LM_w_-PLLA-11k, they are all reversible as temperature cycles are between *T*_c_ to ambient and backward from ambient to *T*_c_. Apparently, cracks occur and become visible only upon cooling from *T*_c_ of fully crystallized PLLA samples; nevertheless, the crack patterns are intimately guided by the crystal assembly in the spherulites.

For controlled cooling rates (10~90 °C/min), cracks appear only slowly and progressively, one after another, to a final stable state. For the ring-banded spherulites in LM_w_-PLLA crystallized at *T*_c_ = 125 °C, there are no cracks visible at *T*_c_. Conversely, as the 125 °C-crystallized PLLA is further cooled at a programmed rate first from *T*_c_ to an intermediate temperature = 60 °C, a few radial segmental cracks first appear, but not all cracks. With further cooling to a lower *T* = 40 °C, the twin-circumferential cracks start to appear sequentially, but not yet in full circles of cracks. Only when the samples are finally cooled to 26 °C and held for a long enough time, all three or four types of cracks (twin circumferential circular cracks, short radial cracks, and tri-star cracks on nuclei center) eventually are all visible. These facts suggest that cracks are a kinetically slow process, much lagging behind temperature equilibration. Contraction by temperature cooling is at work, but much slower detachment in the inter-lamellar interfaces takes time to go to full scales of cracking. The fact of slow appearance of PLLA cracks with cooling is in line with the experimental observation of bulk properties that melt-cast PHB sheets normally become brittle on storage [15]. Thus, simple contraction by CTE directional differences alone, without taking into account the lamellae assembly in spherulites, may not be adequate in accounting for the slow process of formation of cracks.

AFM depth profiles in the PLLA spherulites of unconstrained vs. constraint PLLA samples were analyzed using AFM for measuring the crack depths in PLLA crystallized at various *T*_c_ (120–135 °C) [29]. The crack depth increases with *T*_c_ to a maximum (*T*_c_ = 125 °C), with a depth of 650 nm or 457 nm for the uncovered and covered samples, respectively), and then it decreases slightly with further increase of *T*_c_ beyond 125 °C for uncovered or covered PLLA samples alike. The crack depth, however, does not propagate through the entire film thickness, and is limited to the upper surfaces.

Different materials (glass vs. other substrates) have been examined as top covers for polymer films to crystallize, and then cooled to ambient. Same optical birefringence patterns and same cracks are resulted and it does not matter whether glass slides, polyimide (PI) or Teflon films are used as the materials of top covers, as long as film samples are covered during crystallization. The differences of the top-cover materials do not seem to effect a change in the ring-banded patterns or cracks in spherulites in PLLA [29]. Ring bands in spherulites are always present in crystallized PLLA films if the films are confined between two surfaces, and cracks are resulted upon cooling. Although the material of the substrates (or top covers) may not be an influencing factor for formation of the ring-banded spherulites in PLLA, the cover confinement on sample films imposes an inherent change in lamellae assembly in the spherulites, which further influence the crack patterns. Stress alone by the cover on samples may create some additional stresses, but it may not be sufficient to account for the complex changes in crack patterns between samples with covers or no covers. In addition, some argued that secondary crystallization by holding long time at T_c_ might create further stress for cracking [12,13]. However, it should be emphasized that no cracks in PLLA appear during isothermal crystallization at *T*_c_ for any long time. Apparently, secondary crystallization-induced stress is not the influencing factor for the formation of cracks in PLLA samples. Secondary crystallization does have possibility of generating more lamellar branches grown from main lamellae; nevertheless, these finer branches would not further alter the crack patterns.

**Figure 4:**
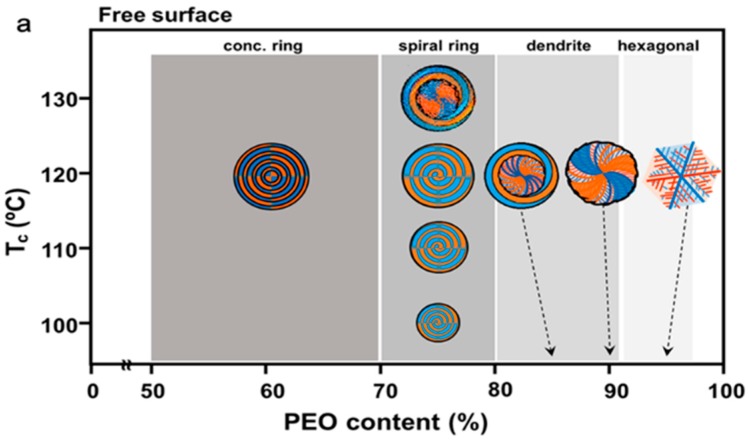

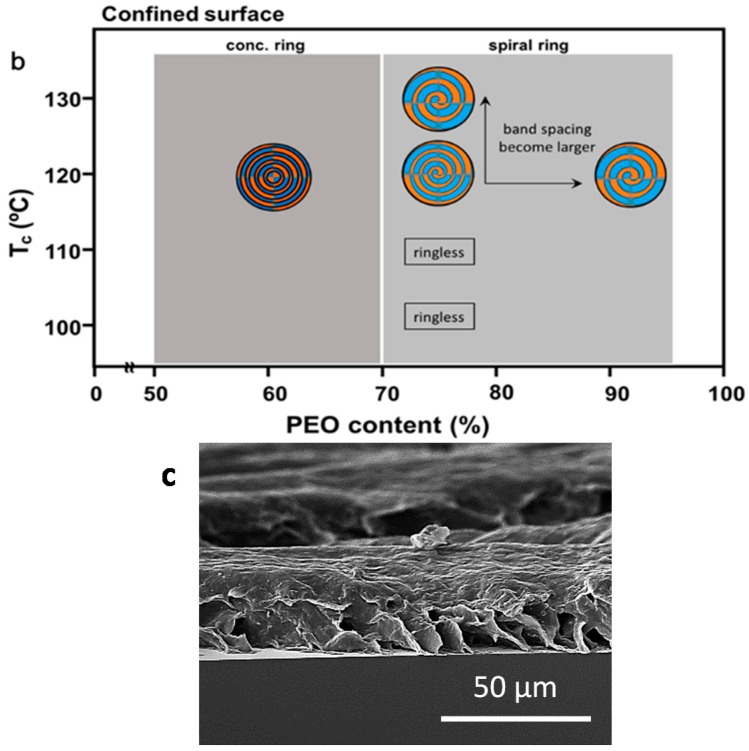
Schemes of PLLA spherulites in crystallized PEO/PLLA blend film samples with (**a**) free top surface and (**b**) top confined films, of different compositions and crystallized at a range of temperatures (100–130 °C); and (**c**) SEM graph of PEO/PLLA (83/17) blend without top-cover confinement, crystallized successively at *T*_c_ = 120 °C, then cooled to room temperature. Interior lamellae showing orientation-induced cracks and interfaces. Reproduced with permission from Ref. [11].

Woo et al. [11] have shown a thorough study on mechanisms of alteration of lamellae assembly in spherulites as PLLA samples [blended with diluent poly(ethylene oxide) (PEO)] crystallized with top-confinement vs. top-free samples. It is the changes in the lamellae assembly (owing to confinement) that induce significant changes in patterns of cracks. Figure 4 shows the ring band patterns in PLLA/PEO blend vary with *T*_c_, diluent composition, and (Figure 4a) top confinement or (Figure 4b) free top surface on sample films. The interplay of diluent PEO composition, confinement, and *T*_c_ leads to quite complex influence on the ring band patterns in crystallized PLLA spherulites. With the presence of high contents of PEO in blends, ring-banded spherulites are common in top-covered sample films at all *T*_c_s; while ringless spherulites are more likely in unconfined films at low *T*_c_s (lower than 110 °C). To sum up, the ring-band patterns vary from circular concentric, to counter-clockwise spirals, then S-shape wheels, and finally to hexagonal-dendritic spherulites. Interestingly, all these geometric shapes of cracks have been all reported in PLLA crystallized films under various conditions. Figure 4c shows SEM graph of PEO/PLLA (83/17) blend (free top surface), crystallized at *T*_c_ = 120 °C, then cooled to RT. The exposed interior PLLA lamellae show numerous interfaces and cracks resulted from lamellar branching or twisting to different orientations. Apparently, the variation in the optical birefringence patterns in PLLA spherulites reflects a change in the interior crystal lamellae assembly; thus, the inter-lamellar interfaces also vary, leading to different crack forms when spherulites are cooled.

**Figure 5:**
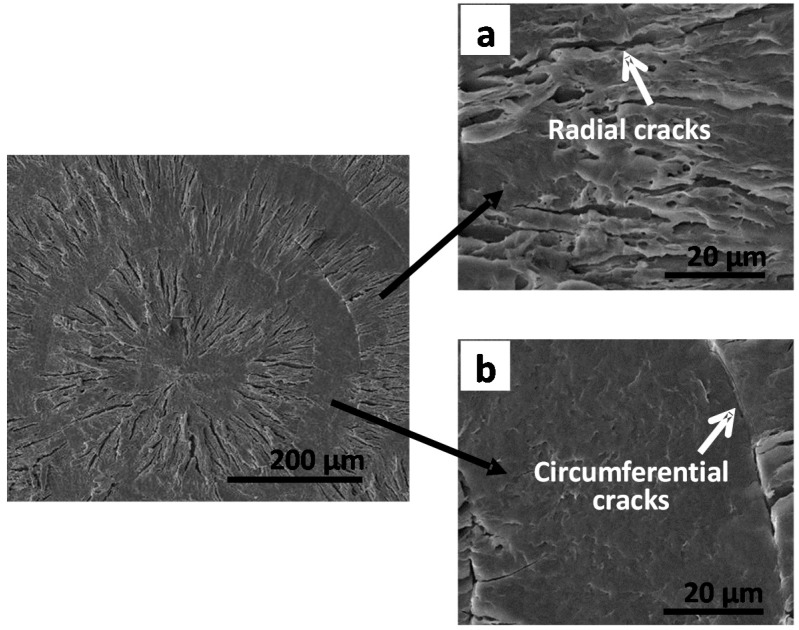
SEM graphs of LM_w_-PLLA-11k isothermally crystallized at 125 °C with a top cover (constrained) showing: (**a**) band with radial cracks; and (**b**) band with twin circumferential cracks in spherulites of PLLA isothermally crystallized at 125 °C and etched with p-dioxane. Reproduced from Ref. [29] with permission from Springer.

Ring bands and lamellar patterns in crystallized LM_w_-PLLA(11k) could be easily discerned using POM via birefringence patterns, but the detailed lamellar textures associated with the ring bands were hidden and seemingly covered by a top layer that prevented detectability using SEM without etching the samples. The crack patterns in crystallized and cooled PLLA could be further observed by SEM or AFM in greater details. Figure 5 shows SEM analysis on samples of p-dioxane-etched LM_w_-PLLA crystallized with ring bands and cracks (short-radial and twin circumferential cracks). Objective is to show correlation between the cracks and interior lamellae assembly. The SEM graphs show discontinuous optically bright (rough) and dark (smooth) bands in the spherulites of LM_w_-PLLA isothermally crystallized at 125 °C, in which the circumferential cracks act as boundaries between these two bands. Short radial cracks are never seen in the dark (smooth) bands and are located only on the bright (rough) band, which separate the radially oriented lamellar bundles in the bright band. The cracks remain the same before and after p-dioxane etching, but the lamellar textures in the etched PLLA sample are fully exposed after etching. The short radial cracks are parallel to the interfaces of the radial lamellae; furthermore, the circumferential cracks are the interfaces of the radially-oriented lamellae and another group of lamellae whose textures are dramatically different from the radially-oriented lamellae. The perfect matching between the spacing of circumferential ring bands and inter-distance of circumferential cracks in the LM_w_-PLLA spherulites melt-crystallized (films confined between two glasses) at *T*_c_ = 125 °C suggests an intimate correlation between the cracks and interior lamellae assembly [29].

## 4. Cracks in PLLA Spherulites Crystallized with sc-PLA as Nuclei or Templates

**Figure 6:**
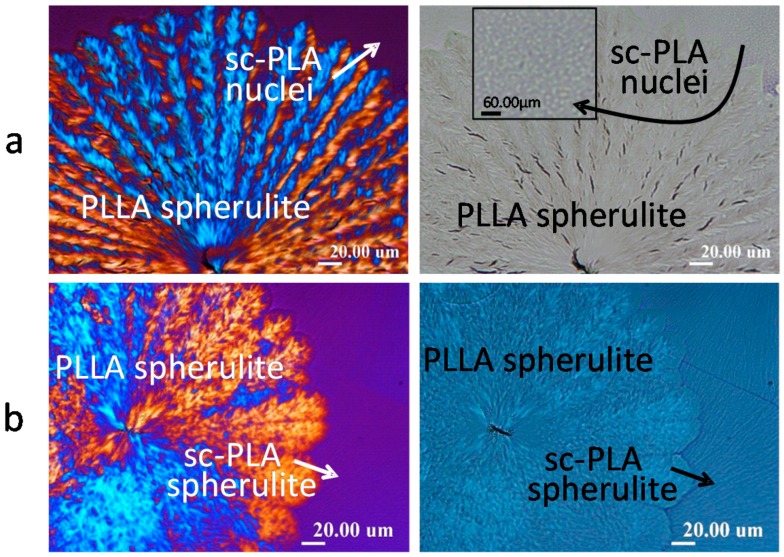
POM (**left**) and OM (**right**) morphology of PLLA/PDLA (90/10) samples subjected to (**a**) *T*_max_ = 190 °C then *T*_c_ = 140 °C, in comparison to (**b**) *T*_max_ = 240 °C then crystallized at *T*_c_ = 140 °C, cooled to ambient and melted/recrystallized again at *T*_max_ = 190 °C and *T*_c_ = 140 °C. Reproduced with permission from Ref. [19].

One of our recent studies has proven that introduction of stereocomplex poly(lactic acid) (sc-PLA) crystals as small nuclei or sc-PLA spherulites into PLLA has great influence on crack patterns or reducing/eliminating the cracks via altering the inter-phase boundaries in crystallized mixtures of poly(l-lactic acid) (LM_w_-PLLA-11k) blended with poly(d-lactic acid) (HM_w_-PDLA-124k) as a non-racemic 90/10 mixture. Figure 6 shows POM morphology and cracks of PLLA/PDLA of non-equimolar (non-racemic) mixture (90/10) subjected to *T*_max_ = 190 °C and *T*_max_ = 240 °C, respectively, then both crystallized at *T*_c_ = 140 °C and cooled to ambient. By heating the mixture at *T*_max_ = 190 °C or 240 °C, respectively, the unmelted sc-PLA tiny residual crystals serve as heterogeneous nuclei to induce later-crystallized LM_w_-PLLA into straight-stalk dendritic spherulites with plenty of radially oriented cracks. The radial cracks, though bent and twisted with lamellae, are apparently located on the inter-phases or interfaces of these generally radial-oriented dendrites. On the other hand, by heating the mixture at *T*_max_ = 240 °C, the PLA-complex crystals were completely melted and upon lowering temperature to *T*_c_ = 140 °C, sc-PLA first crystallize into Maltese-cross spherulites that serves as templates for later-crystallizing LM_w_-PLLA to attach. Co-crystallization of LM_w_-PLLA-11k with the pre-existing sc-PLA templating spherulites tends to be as compact as that of sc-PLA spherulites, thus showing virtually no cracks at all. In general, the sc-PLA spherulites as templates for later-crystallizing LM_w_-PLLA-11k to crystallize (Figure 6a) can suppress the occurrence of stressed interphases in dendritic lamellae in the LM_w_-PLLA-11 spherulites crystallized with tiny sc-PLA nuclei (Figure 6b). One can see that LM_w_-PLLA-11k so crystallized with a small amount of PDLA (10 wt %) in either case of Figure 6a,b develops completely different spherulitic morphologies and correspondingly different crack tendencies as those seen in neat LM_w_-PLLA-11k (shown earlier in Figure 1 and Figure 2). Note that the background thin dendritic thin crystals (indicated by arrows) underneath the strongly birefringent PLLA dendrites were judged to be sc-PLA (minor 10–20 percentages in the blend) for two proven reasons: (1) they are very thin and very weakly birefringent that are formed much more quickly at *T_c_* = 140 °C than the later-forming PLLA dendrites (strongly birefringent); and (2) these thin background dendritic crystals did not melt upon re-heating to 170 °C, where the strongly birefringent PLLA dendrites melt completely. They finally melt only after heating to 230–240 °C (*T*_m_ of sc-PLA).

**Figure 7:**
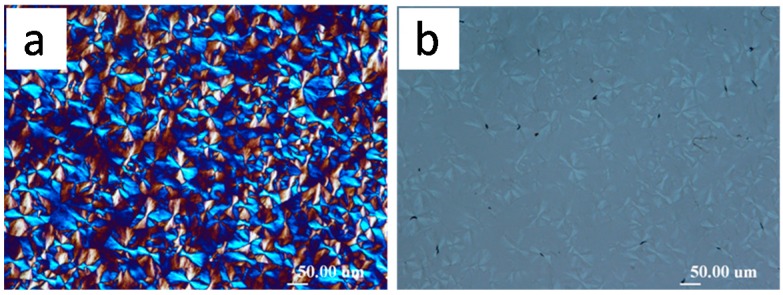
POM (**a**) and OM (**b**) micrographs showing no cracks (except for tiny cracks on nuclei centers) on small ringless spherulites of PHB/LM_w_-PLLA-11k blend isothermally crystallized at *T*_c_ = 100 °C, with top cover, then cooled to ambient. Reprinted with permission from Ref. [20]. Copyright (2011) American Chemical Society.

Similarly, if PLLA is blended with another polymer that is slower-crystallizing than PLLA, then PLLA will serve as templates for the slower-crystallizing polymer to crystallize sequentially. Example is demonstrated with a 50/50 mixture of LM_w_-PLLA with poly(4-hydroxy butyrate) (PHB), as reported in one of our recent studies [20]. Crystallization of poly(3-hydroxybutyrate) (PHB) was performed on previously crystallized templates of poly(L-lactic acid) (LM_w_-PLLA-11k) in mixtures of two semicrystalline polymers, PHB/PLLA (50/50) blend, and cracks, lamellar assembly, and ring band patterns, were analyzed. The PHB/PLLA (50/50) blend was crystallized first at *T*_c_ = 100 °C (ringless spherulites) or 120 °C (ring-banded spherulites), then cooled to ambient, where PHB further crystallizes and cracks show up. At *T*_c_ = 100 or 120 °C, only LM_w_-PLLA-11k can crystallize while PHB remains as an amorphous liquid; then at ambient temperature, the PHB component can crystallize by following the preformed PLLA lamellar crystals as templates. Figure 7 shows, when crystallized at *T*_c_ = 100 °C and cooled, there are no ring bands in spherulites and no cracks are seen, except for small tri-branch cracks on the nuclei centers. At this low *T*_c_ = 100 °C, pure LM_w_-PLLA-11k would also display no ring bands and no cracks. Thus, the LM_w_-PLLA/PHB (50/50) behaves similarly to neat LM_w_-PLLA-11k, as the later-crystallizing PHB simply crystallizes at ambient by following the pre-crystallized PLLA templates.

**Figure 8:**
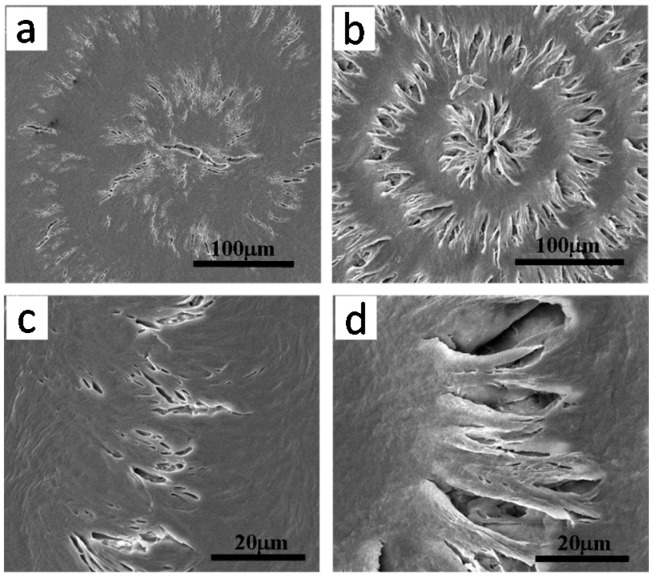
SEM micrographs for PHB/LM_w_-PLLA (50/50) blend samples (*T*_c_ = 120 °C): (**a**) unetched; (**b**) solvent-etched; and (**c**,**d**) zoom-in SEM graphs of the bright ridge band in ring-banded spherulites of Graphs (**a**,**b**), respectively. Reprinted with permission from Ref. [20]. Copyright (2011) American Chemical Society.

By comparison, Figure 8 shows when LM_w_-PLLA-11k/PHB (50/50) blend is crystallized at *T*_c_ = 120 °C and cooled, there are concentric ring bands in the spherulites and short radial cracks are seen, which are similar to those in neat LM_w_-PLLA-11k. Again, the LM_w_-PLLA/PHB (50/50) blend behaves and displays spherulitic morphologies and crack patterns similarly to neat LM_w_-PLLA-11k. The SEM graphs in Figure 5 for pure LM_w_-PLLA-11k at *T*_c_ = 125 °C with a top cover show morphology and cracks that are almost identical to the LM_w_-PLLA/PHB (50/50) blend. The only difference is that there are no twin circumferential cracks in the LM_w_-PLLA/PHB (50/50) blend. The short radial cracks, as clearly shown in the SEM graphs, are resulted from the interfacial boundaries between the radial-oriented lamellae.

**Figure 9:**
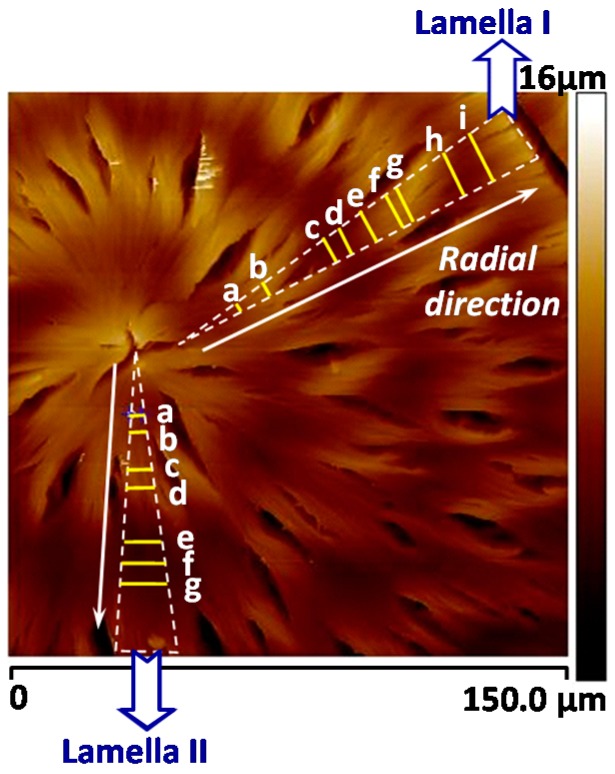
AFM height image on water-etched spherulites of PEO/PLLA (50/50) blend crystallized at *T*_c_ = 110 °C and cooled to ambient. Two discrete macro-lamellae (I,II) marked for height profile analysis. Reprinted from Ref. [33]. Copyright (2012), with permission from Elsevier.

To prove universal behavior, LM_w_-PLLA-11k is blended with another polymer, poly(ethylene oxide) (PEO), at 50/50 ratio and crystallized at *T*_c_ = 110 °C, and then cooled to ambient. Again, the spherulite morphology and short radial cracks in the PLLA/PEO blend are quite similar to those in pure LM_w_-PLLA-11k. To analyze the height profiles of the lamellae parallel to the radial cracks, AFM characterization and results are shown in Figure 9, which reveals that there are concentric ring bands and short radial cracks in the spherulites, almost identical to those in neat LM_w_-PLLA-11k. The novel AFM analysis on the banded and cracked PLLA spherulites shows that there is no spiral lamellar plate but the lamellae wave up and down to create corresponding ridge and valley bands. Short radial cracks are located on the bulged concave portions, where radially-oriented lamellae show inter-lamellar detachments into cracks.

Recently, Sasaki et al. [34] articulately reported and analyzed spiral crack patterns observed for spherulites of poly(L-lactic acid) (*M*_w_ = 210k, l-isomer content: 98%) melt-crystallized at *T*_c_ = 110 or 120 °C. The as-cast PLLA film was approximately 5–25 μm. They found from the cross-sectional SEM images of as-quenched spherulitic plates of PLLA specimens that the cracks span from the top surface to the PLLA/substrate interface. In addition, they found numerous concentric circular ring-type cracks or right-handed and left-handed spiral cracks in the crystallized spherulites. After etching treatments, delamination (from substrates) of PLLA spherulites occurs. As a result of the delamination, deformation or fragmentation of the spherulite occurs on several occasions; in the severest case, the center part of the detached spherulite is lost, leaving an open-circle ring of the PLLA spherulites. For the spiral-shape cracks, Sasaki et al. [34] conducted sophisticate analyses of plotting the distance r from the spherulite center to the trajectory of the spiral against the rotation angle θ, and attempted to model the CTE contraction patterns using an “Archimedean spiral” (in polar coordinates: r = a + bθ), including the energy released during cracking. They concluded that they “have demonstrated characteristic spiral crack patterns on polymer spherulites for the first time”, and “there is no solid evidence that the observed spiral pattern originates from any periodic lamellar assembled structures”.

Sasaki et al. [34] claimed that the present PLLA spherulites exhibited a non-banded structure, and thus no direct correlation between the crack pattern and the spherulitic structure was suggested. However, they also speculated (yet unproven) that the type and handedness of the crack pattern depends on the geometry of an initial crack, which is governed by the lamellar structure in the center part of spherulite. This is a key point. One of our recent wok on lamellar assembly in chiral PLLA or PDLA spherulites has given a clue [35]. With thicker PLLA specimens, the ring banded patterns may be hidden, becoming optically invisible, owing to repeated covering of later-grown crystals; nevertheless, the crystal assembly near the nuclei center remains the same. We have proven recently [35] that for thicker PLLA samples (diluted with high contents of PEO for convenience of etching out to expose the skeleton PLLA lamellae), growth begins with nucleation as PLA hexagonal crystals, but eventually, growth continues to take a final spiral pattern. Since the growth rate of spherulites is higher in thicker films, bending occurs with a larger curvature as crystals radiate out from the hexagon center and such larger curvature combine with faster growth induces the formation of spiral growth. The growth from the nuclei center is originally spiral in both counter-clockwise and clockwise directions. Nevertheless, they may not all grow equally, and if one dominates the other, the spiral can become either clockwise or counterclockwise. Such lamellar assembly near the nuclei center might be submerged in thicker PLLA specimens; yet, it provides initiation for crack propagation and patterns. We believe that, regardless of the visibility of ring band patterns in PLLA as the optical bands tend to be hidden or disrupted in thicker samples, the inherent lamellae assembly, either near the nuclei center or in final spherulites, is accountable for the spiral cracks as reported in Sasaki and coworkers’ work on cracks. Furthermore, if one inspect the spiraling ring-band patterns that we have revealed in crystallized PLLA as discussed in Figure 4, it is apparent that there exists apparent correlation between the ring bands and spiral crack patterns reported by Sasaki et al. [34], suggesting that lamellae assembly in spiral ring bands and interfaces between successive bands with hidden discontinuity might initiate the crack patterns to be similarly in spirals (either clockwise or counterclockwise senses).

## 5. Correlation of Cracks with Hexagonal Ring Patterns and Disappearance of Rings in Increasingly Thicker Specimens

**Figure 10:**
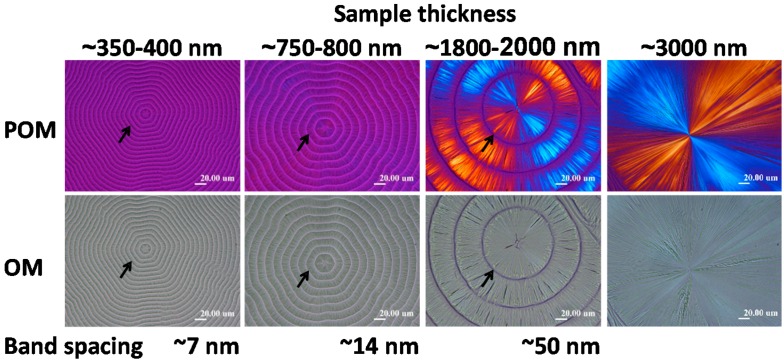
POM (**top**) and OM (**bottom**) micrographs of LM_w_-PLLA-11k melt-crystallized at *T*_c_ = 112 °C at various sample thickness as indicated on the top of the graphs Reproduced with permission from Ref. [36].

Cracks or their patterns in crystallized polymer films upon cooling, as discussed, can be dependent of the film thickness. Similar dependence can also be seen in ring band patterns of crystallized polymers. Woo et al. [36] investigated the thickness dependence of ring-band patterns in LM_w_-PLLA-11k films, which were all crystallized at same *T*_c_ = 112 °C, but thickness of sample films were varied systematically from 400 to 3000 nm. Figure 10 shows LM_w_-PLLA-11k [36] melt-crystallized at *T*_c_ = 112 °C of films at various sample thicknesses, 400 nm, 800 nm, 2000 nm, and 3000 nm, as indicated on the top of the graphs. Concentric ring bands (low birefringence) of hexagonal shapes are present in films of 400 and 800 nm. The bottom graph in Figure 10 shows the AFM image of the hexagonal ring bands in 112 °C-crystallized PLLA-11k film of 800 nm (and cooled to ambient), revealing apparent deep crevices between the successive hexagon-shape bands. The band spacing apparently increases with film thickness almost linearly. For PLLA films of 2000 nm, the birefringent ring bands are strong in optical birefringence and quite wide (~40 µm in band width) separated with a very narrow extinction, which according to corresponding AFM height analysis (not shown here) on same sample is a sharp drop toward the glass substrate. For films of thickness = 3000 nm or thicker, the optical bands are no longer seen. To sum up, the discontinuity between the ring bands (regardless of hexagonal or circular shapes) are apparent for film thickness of 400, 800, and 2000 nm. The thicker LM_w_-PLLA-11k film of 3000 nm crystallizes to ringless spherulites; nevertheless, as discussed earlier, interfaces and ring bands may be covered up and hidden, and possibility still exists that they are able to initiate cracks of either circular or hexagonal shapes upon cooling.

**Figure 11:**
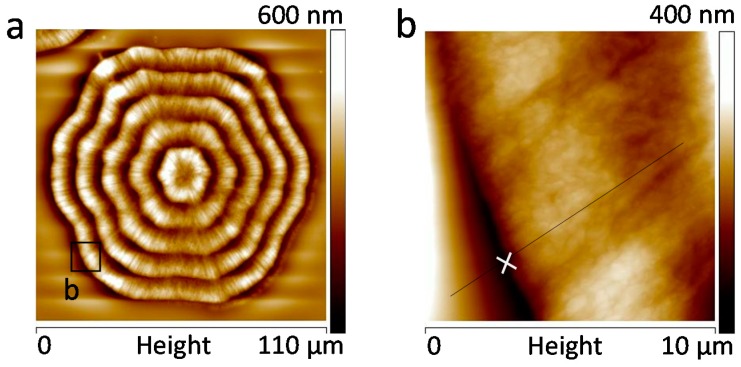

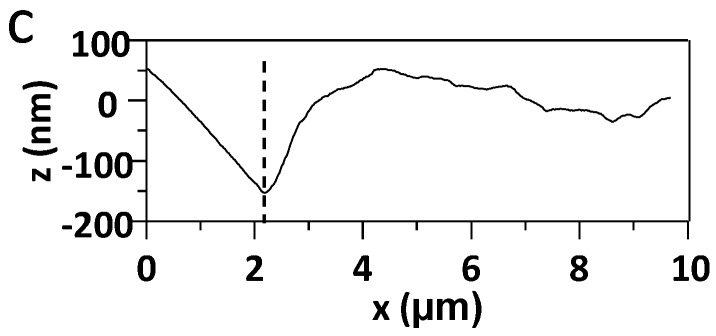
AFM height images of hexagonal-shaped rings in LM_w_-PLLA-11k spherulite melt-crystallized at *T*_c_ = 112 °C: (**a**) full image; (**b**) zoomed-in image; and (**c**) height profile.

Furthermore, Figure 11 shows zoom-in to inter-band zone using AFM analysis (AFM height profile and phase image), which clearly reveals inter-band discontinuity in the hexagonal-shaped rings (for film thickness = 800 nm) in LM_w_-PLLA-11k spherulite. The depth of inter-band depletion zone is at least 200 nm out of the film thickness of about 800 nm. The graphs are shown such that the radial growth direction is from right to left-hand side. Apparently, if cracks are to propagate, they will naturally follow the inter-band crevices, which are of a hexagon shape (as shown earlier in Figure 10).

**Figure 12:**
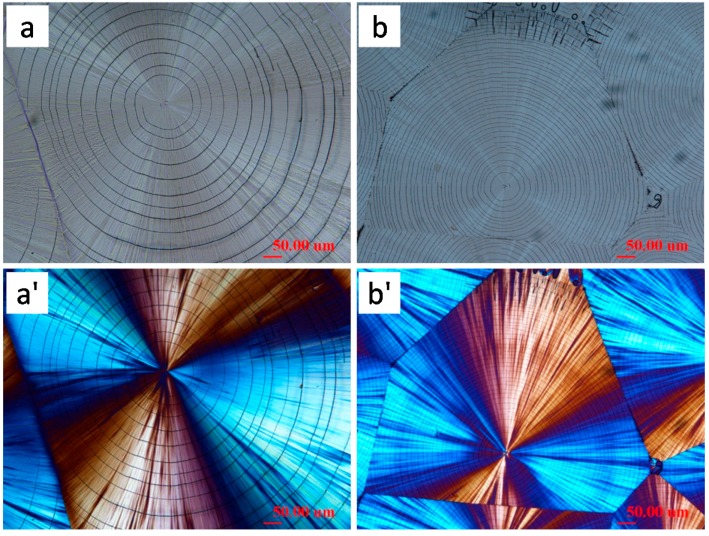
OM (**top**) and POM (**bottom**) micrographs of HM_w_-PLLA (152k) melt-crystallized at *T*_c_ = 135 °C showing cracks and optical birefringence in crystallized and cooled samples: (**a,a’**) sample uncovered; and (**b,b’**) sample covered with top glass.

There appears to be some proven correlations between the crystal assembly as revealed in optical birefringence and cracks; however, further compounded the problem is that cracks tend to be influenced by many factors: film thickness, *T*_c_, top glass cover, cooling speed and time (slow cooling vs. dipping in liquid nitrogen), molecular weight of polymers, etc. In several situations, correlations may be difficult or impossible to assess. An interesting example is demonstrated here. Figure 12 shows cracks in OM micrographs and cracks/birefringence patterns of HM_w_-PLLA-152k melt-crystallized at *T*_c_ = 135 °C (sample films were uncovered vs. covered for comparison). The crack behavior of this HM_w_-PLLA-152k grade is apparently different from that of LM_w_-PLLA-11k (Figure 1 and Figure 2). At *T*_c_ = 135 °C, HM_w_-PLLA-152k crystallizes into only ringless spherulites, with or without glass covers on sample films. After crystallization at *T*_c_ = 135 °C and upon cooling, cracks in both PLLA samples (with or without top glass cover) develop extensively. Although both samples develop cracks, there are noticeable differences in the main characteristics for the uncovered films (Figure 12a) and covered films (Figure 12b). The crack shape and density are dramatically different between the samples crystallized and cooled with free surfaces vs. samples with top-cover glass. For the uncovered HM_w_-PLLA films (Figure 12a), the cracks resemble the Archimedean continuous spirals but are of a hexagonal shape near the nuclei center, gradually transforming into circular spirals as they traverse to external peripherals. The hexagon or circular spirals are mostly continuous within the spherulites. This is quite interesting feature in that the cracks are not only spiral, but also hexagon shape. On the other hand, for covered HM_w_-PLLA films (Figure 12b), the cracks in the covered films are mostly concentric circles and much denser (than the uncovered films); in addition, most of the cracks are discontinuous and tend to be shattered into broken pieces. These facts in the covered PLLA samples suggest that additional stresses by top glass cover on crystallized and cooled PLLA spherulites may cause additional cracks.

## 6. Cracks in Spherulites of Ductile Polymers in Comparison with Brittle Polymers

Isotactic polypropylene (iPP) is a ductile polymer, and usually its spherulites do not develop cracks upon cooling as those seen in PLLA or PHB. However, for a huge isotactic polypropylene (iPP, *M*_w_ = 260k) spherulite (under uniaxial tension deformation), Nitta and Takayanagi reported [37] that circumferential arc-shape cracks appeared in the polar zone of spherulite while radial craze-like fracture appeared in the equatorial region (perpendicular to the stretching direction). With respect to changes in the dominant lamellae in the iPP spherulite (number of tangential lamellae in all negative-sign at *T*_c_ = 130 °C or mixed neg/pos-sign spherulites at *T*_c_ = 145 °C), the crack patterns also change correspondingly. An alternative study by Nowacki et al. on iPP [38] reported that upon simple cooling of iPP (without imposed tension), only radial cracks may occur near the spherulites boundaries (weak spots) during cooling process. That is, the cracks in iPP are mainly in inter-spherulitic areas, but not within the spherulites, and they claimed that local stresses or holes weaken the material; hence, the areas occluded during crystallization constitute “weak spots” of the spherulitic structure [38]. Unlike the ductile iPP spherulites, brittle spherulites such as PLLA and PHB, may have more substantial “weak spots” within the large spherulites, leading to corresponding intra-spherulitic cracks.

Once again, these comparative facts on iPP further demonstrate that the crack patterns, either in ductile iPP or relatively brittle PLLA or PHB spherulites, can be governed by the pre-assembled lamellae and their interfaces in the spherulites. Similarly, for large spherulites such as those in brittle PLLA or PHB, weak spots are not just in inter-spherulite boundaries, but also in intra-spherulites, where boundaries/interfaces among the lamellae may exist in sporadic or regular patterns within the spherulites.

## 7. Cracks in PHB Spherulites

Barham and Keller [5] were among the early pioneers who investigated cracks and brittleness in PHB in 1986. They briefly discussed about the possible correlation between cracks and “pre-existing microstructures” in lamellae of spherulites, and modes of fracture of bulk PHB. Barham and Keller [5] believed that the brittleness of PHB sheets is due to preexisting cracks found within the spherulites, and they also proposed that ductility might be achieved by “healing” of the cracks, or by crystallizing to lead to crack-free sheets. However, they emphasized that the mechanisms of crack formation remain open issues. Barham and Keller [5] at that time recognized that there is the possibility of a link between the radial cracks and the crystal growth regime, which clearly invites much further work. Clearly, they strongly felt that there might be correlations between the cracks and lamellae microstructures. However, at that time, they were not sure about how to establish correlations for details of mechanisms, partially owing to at that time they used only tools of optical microscopy (and mechanical tests of bulk properties), which limited probing into the hierarchical microstructures in spherulites. Later in 1989, Keller et al. [14] further investigated cracks in PHB and found that two types of cracks are at two different temperatures of crystallization: radial cracks at low The radial cracks occur at low crystallization temperature (*T*_c_ = ~80 °C), and circumferential cracks on cooling spherulites crystallized at higher temperature (*T*_c_ = ~100 °C). Using X-ray analysis, they measured the variation of the Bragg spacings in (110) and (200) planes with the temperature, which increase with different slopes in these two directions. They proposed that different radial and circumferential thermal expansion coefficients (CTE), which generate large internal stress, leading to the cracks. However, we would like to caution here two critical points that might not have been accounted in that study. One is that PHB spherulites are not composed of all single-crystal lamellae with monotonous variations in directional lattice planes. Secondly, crystallization of PHB at *T*_c_ = 80 vs. 100 °C can lead to entirely different lamellar microstructures (e.g., Maltese-cross vs. ring-banded spherulites, respectively, at 80 and 100 °C).

Without examining the detailed interior microstructures of PHB spherulites at these two temperatures, it may be premature to attribute to differences in CTE in two directions. However, Keller et al. [14], in the study of cracks in PHB, did correctly discuss about a case of polyethylene in another study on PE [39] to state: “However, in that case of polyethylene (PE) spherulites, the difference in the expansion coefficients is NOT accounted for by cracking but by the twisting, or radiating lamellae, as manifested by changes in the periodic extinction band spacing” (by referring to the similar extinction rings discussed earlier in Figure 1 and Figure 2 for PLLA). Cracks in polymers are a result of directional contraction upon cooling; nevertheless, microstructures in spherulites developed at specific *T_c_*s should be taken into consideration for fuller account.

The fact that the microstructures of lamellae in spherulites should be accounted for in analyzing cracks in spherulites can also be envisioned by an article by Hobbs et al. [40] on AFM analysis on polymer spherulites. They have demonstrated, using AFM imaging, varieties of crystals in growing spherulites during initial growth and secondary growth during subsequent cooling; and these crystal features in spherulites include: shish-kebab, screw dislocations, branching lamellae from screw dislocations, etc., in PE or iPP. Such assembly of polycrystals, in either ring-banded or ringless spherulites alike, inevitably involves amply interfaces and possible weakened boundaries. These facts evidently suggest that polymer spherulites are not composed of only continuous single-crystal lamellar plates monotonously radiating out from nuclei centers. Inter-lamellae boundaries, in radial or circumferential directions, should be accounted.

**Figure 13:**
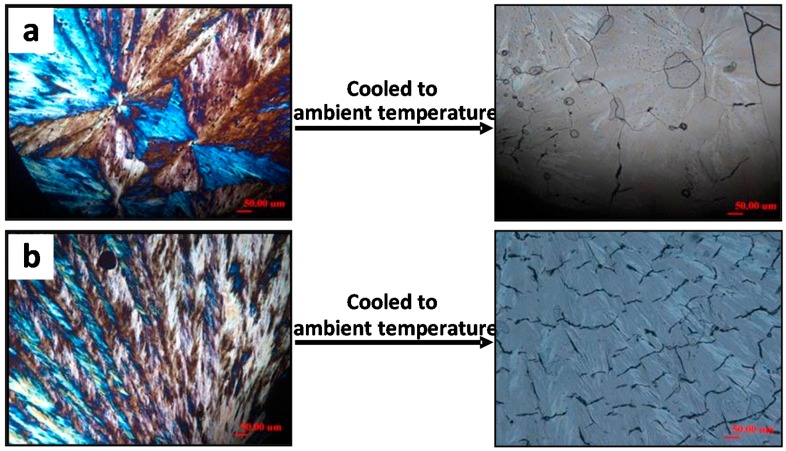
Irregular tangential cracks and ringless spherulites as revealed in POM (**left** column) and OM (**right** column) for LM_w_-PLLA-11k isothermally crystallized at 130 °C: (**a**) glass substrate untreated but glass cover pre-treated with release agent; and (**b**) both glass substrate and cover treated with release agent (scale bar = 50 μm in all graphs).

Crystallization of polymer films on substrates is attributed by some investigators to the main cause of stress for cracking. Hobbs et al. [15], in 1996 in another study on cracks in PHB, reported: “We have shown that cracks and crazes formed in spherulites of PHB, during growth and on cooling, are due to constraints imposed by the supporting substrate of the thin film and are therefore unlikely to be of importance in determining the ultimate mechanical properties of the bulk material”. Nevertheless, we want to caution here again that although polymer-substrate contact is indeed a potential site of stress, the substrate alone cannot be the main cause of stress leading to variety of crack forms as seen in LM_w_-PLLA discussed earlier. Cracks including short radial, circumferential, and tri-branch cracks, are seen on the top free surfaces of LM_w_-PLLA, which are far away from the polymer-substrate interface. It is worthy to demonstrate that the substrate-polymer contact and stress is not fully accountable for the cracks, although the contact with substrate (or confinement by top cover) may alter the lamellae assembly of polymers being crystallized. As discussed earlier in Figure 1 and Figure 2, LM_w_-PLLA-11k films covered with a glass cover would display ring bands and corresponding circumferential and short radial cracks when crystallized at *T_c_* = 125~130 °C. However, top glass covers were treated with a release agent (to reduce the contact stresses), and effects of the treated top glass on crystallization and cracks on films were assessed. Figure 13 shows that for the same LM_w_-PLLA-11k crystallized with a glass cover that had been pre-treated with a release agent, the PLLA films displayed ringless spherulites, instead of ring-banded ones. With cooling to ambient, cracks still develop in the ringless spherulites, although the crack forms are different from those in covered films (or normal substrate glass untreated with release agent). These facts suggest that stress between the substrate and polymers alone does not govern the mechanisms of cracks and that substrate interfaced with polymer films might alter the lamellae assembly (ring-banded vs. ringless) and corresponding crack patterns, but cracks may still be present. Effects of surface treatment of the glass substrate or glass cover on spherulite morphology were investigated. Either the glass substrate or glass cover was coated with a release agent. Figure 13 also shows cracks as revealed in POM and OM morphology of LM_w_-PLLA-11k spherulites isothermally crystallized at 130 °C and cooled, where sample films were on (a) glass substrate untreated but glass cover pre-treated with a release agent; and (b) both glass substrate and glass cover were coated with a release agent. Interestingly, no ring bands are seen in either PLLA samples (substrate treated or substrate/cover treated with release agent) when crystallized at 130 °C. By comparison, earlier Figure 1 and Figure 2 already have shown that the PLLA spherulites are distinctly ring-banded if the PLLA films are confined in untreated glass cover and substrate and crystallized at 130 °C. Release agent apparently releases the polymers from such interactions with the substrate, thus relieves the stress; however, cracks are still present in cooled films. Regardless of ring-banded or ringless morphology in release agent-applied substrate or top cover, cracks are still present in the crystallized and cooled LM_w_-PLLA films. Crystallization temperature and top-cover confinement or substrate contact with polymers are several of the main factors affecting patterns of cracks and occurrence of either ring-banded or ringless spherulites of PLLA.

In addition, previous observations have shown that circumferential cracks do not run through the entire thickness of films; actually, most cracks are confined within the upper half of polymer films. The stresses should be maximum at the substrate-polymer interfaces but minimal at the free top surfaces; yet cracks are confined on the top portions of films. Thus, cracks in PLLA cannot be simply or properly interpreted by the convention mechanisms of the stresses generated at substrate interfaces.

**Figure 14:**
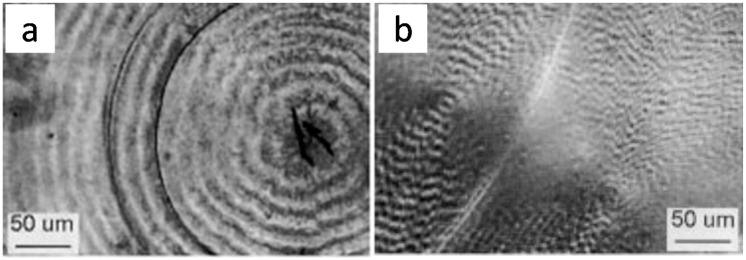
PHB spherulites crystallized at 90 °C from the melt films sandwiched between: (**a**) polyimide slides; and (**b**) poly(ethylene terephthalate) slides. Reproduced with permission from Ref. [16].

Xu et al. [16] reported cracks in PHB crystallized at *T*_c_ = 90 °C and cooled, using alternative substrates (polyimide and poly(ethylene terephthalate) films). As shown in Figure 14, there are two distinct circumferential cracks in PHB films crystallized (at 90 °C) between two polyimide slides (Figure 14a). Although the number of cracks is fewer than that of ring bands, it is obvious that the circumferential circular cracks follow exactly the tracts of the boundaries of the ring bands. In addition, short cracks are present on the nuclei center.

In 2010, Ye et al. [41] also studied PHB, a chiral polymer, to assess the correlation of surface stress with lamellae shapes and chiral senses using theoretical simulations, which show that the distribution of anisotropic surface stresses has considerable influence on the morphologies of lamellar crystals, and that alteration of the direction of surface stresses may change the twisting chirality. Nevertheless, the assumptions of PHB lamellae in that study were based on a continuously spiraling twist (handily adapted from the model of DNA molecular double spirals). Models of continuous spiral twist lamellae (like a spiral ribbon) were regarded as mechanism of periodic optical banding in spherulites since Keller, Keith, and Padden et al. initiated the concepts [22,23,24]. With much more advanced and powerful modern instruments than those available in 1960s, solid proofs on “continuity” and “source for twisting” are still unproven or have been controversially debated to date for nearly fifty years, which suggests that critical pieces of evidence in support of the continuous-twist proposals are yet to be justified with clearer and stronger evidence. Opposite to that, long-time assumed opinion of continuous spirals for banding is expressed by some recent advances. This updated concept was novel for banding mechanisms, and also extremely critical in assessing the cracks in banded spherulites. Woo et al. [25] in 2012 have demonstrated corrugate-board-like structures with apparently inter-ring discontinuity in crystallized and ring-banded poly(ethylene adipate) (PEA). Similarly, in 2013, Schultz [42] claimed in a re-visit of his old 2004 AFM data [43] for poly(3-hydroxybutyrate-co-3-hydroxyhexanoate) (PHBH) ring-banded spherulites that he emphatically reverted the opinion of continuous twisting to that “unusual discontinuity” may exist in the bands, and “what observed here does not accord with any extant models of crystal twisting.” Note here that both PEA and PHBH crystallize into ring-banded spherulites with optical birefringence patterns similar to PHB.

## 8. Cracks in Poly(Trimethylene Terephthalate) (PTT) Ring-Banded Spherulites

Poly(trimethylene terephthalate) (PTT), being a much tougher polymer than PLLA or PHB, shows cracks only in more severe circumstances. Yao and Yang [44] studied cracking in poly(trimethylene terephthalate) (PTT) spherulites grown between two substrates, crystallized at *T*_c_ = 190 °C and cooled to lower temperatures or ambient (120, 90, 40 °C). They reported that PTT spherulites grown between two polyimide and two Teflon sheets showed no cracks, but PTT film grown between two glass sheets lubricated with silicon oil displayed reduced number of cracks but did not totally eliminate cracks. With PTT grown on a single substrate (top free surface) at same *T*_c_ = 190 °C, ring-bands and cracks also appeared. The number of cracks in PTT was low compared to that of spherulites grown between two glass surfaces; however, in thicker PTT films (>30 µm), cracks are still present in crystallized and cooled PTT films. It should be noted that most of cracks in the PTT films roughly follow the ring-band patterns in the crystallized PTT films, although the cracks are more smoothly circular than the zigzag ring bands. This may be rationalized by cracks being likely initiated from some lamellae interfacial boundaries, but propagating more or less in a course not necessarily along the zigzag patterns. By comparison, cracks are never seen when the thickness of PTT films is smaller than 20 um. Again, this fact testifies that stress of polymer-substrate contact alone is not sufficient for cracks. The stress should be decreasingly less for the top surface of thicker PTT films; nevertheless, cracks develop in thicker films with single side of glass substrate, but not in thinner films. They claimed that this behavior in PTT is similar to the case of PHB [15].

**Figure 15:**
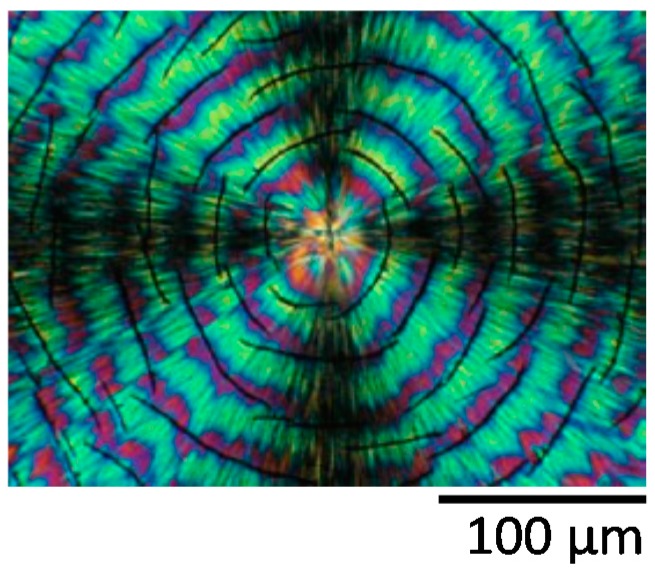
Polarized light micrograph showing cracks and ring bands in PTT spherulite isothermally crystallized at 195 °C and cooled/dipped in solvent. Film thickness is about 20 µm. Reprinted by permission from Macmillan Publishers Ltd Ref. [45], copyright (2008).

Kuboyama and Ogizawa [45] studied solvent-dipping induced cracks in PTT, where PTT spherulites were first isothermally crystallized at 195 °C in films with thickness of about 5, 10 and 20 µm, and the specimens were dipped into chloroform to cause cracking. As shown in Figure 15, the banded pattern and cracks are simultaneously present in PTT spherulites (irregularly ring-banded at *T*_c_ = 195 °C). Like the results we have shown and discussed earlier for the case of LM_w_-PLLA spherulites, the solvent-dipping induced cracks in the crystallized PTT films roughly coincide with the ring-band patterns. The facts reinstates that cracks most likely are initiated in the interfacial boundaries between lamellar bundles as manifested by the ring-band patterns in PTT spherulites. The authors also commented that the cracks in thinner films (Figure 2a,b of Ref. [45]) are less ordered. They also observed that the cracking pattern in PTT films is mostly concentric; however, the number of cracks in the thinner PTT films was more than that in the thicker one. This fact indicates that the spacing between cracks increased monotonically with increase of the thickness of PTT specimen. They also attempted to probe possible correlations between the ring bands and cracks in the PTT spherulites by examining two PTT specimens, one being isothermally crystallized at *T*_c_ = 160 °C (ringless) and the other 195 °C (ring-banded). Ringless PTT spherulite isothermally crystallized at 160 °C, cooled and dipped into solvent, exhibits only one sporadic crack line near the external periphery of spherulites. They concluded that in the PTT banded spherulite, the concentric multiple cracks are formed periodically that correlate well with the banded pattern. However, other than pointing out the correlation, they did not establish how or why the cracks are in correlation with the ring band.

In two of our recent studies dealing with interior lamellae assembly in PTT banded spherulites [46,47], we discovered for the first time that in the ring-banded PTT spherulites, there exists discontinuity and interfacial boundaries between the successive bands in PTT spherulites. This critical finding in ring-banded PTT provides tips of how circular cracks might be initiated, as well as how lamellae crystals are assembled in the ring-banded PTT spherulites. Such finding points a new direction of understanding the cracks not only in PTT, but also in other polymers like PLLA and PHB. In addition, all the trends in the cracks in PTT films of different thickness in the Kuboyama and coworkers’ study [45] are consistently and strikingly in agreement with the variation trends of the ring bands in crystallized PTT of different thickness that we just disclosed recently [46,47].

**Figure 16:**
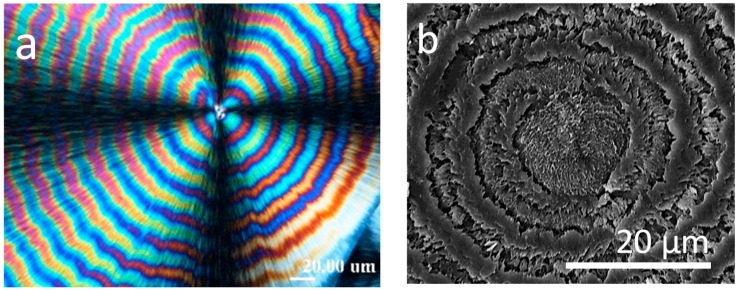
Birefringent rings and crevices between the rings in PTT spherulite crystallized at 165 °C: (**a**) POM graph of virgin PTT; and (**b**) SEM graph of 20 min KMnO_4_-etched PTT. Reproduced from Ref. [47] with permission from The Royal Society of Chemistry.

Note that PTT crystallized at 165 °C has strong birefringent ring bands, but the PTT films does not develop intra-spherulite cracks (except for inter-spherulite impingement cracks) in the PTT films crystallized at 165 °C and cooled. This behavior of lacking cracks in 165 °C-crystallized PTT films is quite different from what observed by Ogizawa and Kuboyama, who reported extensive cracks in ring-banded PTT films (5–20 μm) crystallized at *T*_c_ = 195 °C and cooled/dipped in solvents [45]. Apparently, *T*_c_ and/or solvent dipping is one of main parameters influencing the crack formation in PTT or other polymers. Woo et al. [46,47] studied the ring-banded PTT spherulites (regularly ring-banded at *T*_c_ = 165 °C, and cooled to ambient), and found obvious inter-ring crevices (discontinuity) by etching with potassium permanganate (KMnO_4_). Figure 16 shows results of PTT film crystallized at 165 °C, cooled and etched for 20 min in KMnO_4_ solution. Figure 16a is POM graph of virgin PTT (unetched) with birefringence bands of high orders with extinct Maltese-cross, and Figure 16b shows SEM graph PTT spherulites (permanganate etched) with either spiral crevices. The POM, with the Maltese-cross interference and lack of resolution, may be hard to tell exactly that they are spiral or concentric rings, but the SEM graph tells more clearly that these are spirals originating from the nuclei centers. Etching with potassium permanganate on 165 °C-crystallized PTT with ring bands removed the species of either amorphous matters or stressed crystals at interfaces, exposing the hidden discontinuity between the ring bands. One can see that the rings (in continuous spiral or circular shapes) between the crevices remain intact and not etched by KMnO_4_, indicating the lamellae growing along the circular rings (or spiral rings), with branches growing at nearly perpendicularly. The fact suggests an opposed view to extant models of continuous spiral-twist lamellae radiating and extending from nuclei center to peripheral. These spiral-shaped interfaces between the spiraling lamellae originating from the nuclei center may guide the cracks upon cooling.

**Figure 17:**
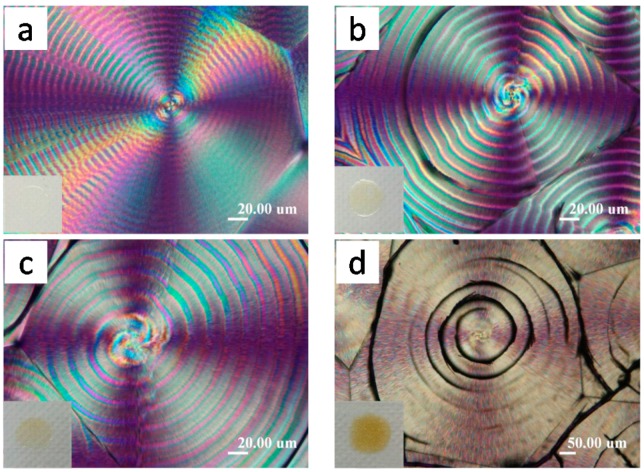
POM graphs showing PTT melt at *T*_max_ = 260 °C for: (**a**) 2 min; (**b**) 5 min; (**c**) 10 min; and (**d**) 30 min, and then quenched to 165 °C for full crystallization before cooled to RT. All the graphs are taken at RT.

Cracks in spherulites occur immediately upon quenching to ambient. In PTT ring banded spherulites, when cracks develop, they always follow the tracts of the ring-band patterns. Figure 17 shows POM graphs for PTT films (ca. 40 μm) that were melt at *T*_max_ = 260 °C for 2, 5, 10, and 30 min, quenched to *T*_c_ = 165 °C for full crystallization and cooled to ambient RT. Interestingly, the ring bands are of a counterclockwise spiral; the cracks, though not in the same number of the ring bands, are also of the same counterclockwise spiral. The spiral optical ring bands are slightly distorted near the core (see POM graph at 10 min); similarly, the spiral cracks are also distorted on the same spots (see OM graph at 30 min).

## 9. Conclusions

Several common factors are identified for influencing the cracks in PLLA, PHB, or PTT: *T*_c_, film thickness, *M*_w_, ductility, spherulite patterns of ring band or dendrite patterns at *T*_c_, prior crystals as templates, growth/contraction under constrained vs. unconstrained (covered with glass or not) conditions, etc. Even cooling speed and time (slow cooling vs. dipping in liquid nitrogen) might influence the extent of cracking. Spherulite size is also associated with cracks. Different *T_c_* alters not only the sizes but also the birefringence patterns of spherulites, and cracks in PLLA are spherulite-size dependent. Apparently, it takes a critical spherulite size for formation of cracks. For small spherulites (<1 µm) or ductile spherulites, cracks are less likely to develop. Film samples crystallized with a top glass cover usually develop more extensive cracks, or entirely different patterns of cracks, than do ones with free top surfaces.

All the crack patterns ever reported in the literature include: (1) tri-branch cracks; (2) short radial cracks; and (3) circumferential long cracks. These may assume four major patterns: (a) circular and concentric; (b) clockwise or counterclockwise spirals (similar to Archimedean spirals); (c) hexagonal shapes; or (d) hexagonal in inner portion, but circular in outer portion. The first two types of cracks (tri-branch and short radial types) are apparently a result of easy cracking along the parallel-oriented lamellae (or lamellar bundles) with interfaces. The long and circumferential cracks (circular or hexagonal shapes) are often coinciding with the ring patterns commonly seen in ring-banded spherulites. The striking similarity between the crack patterns and ring bands in spherulites suggests a possibility of correlations between the cracks in lamellae assembly in the ring-band spherulites. Circumferential cracks tend to follow the same tracks of lamellae packed in the banded spherulites. Crack formation and types of cracks in ring-banded spherulites are intimately associated with the crystal orientation, and patterns of crystalline lamellae and spherulites.

Updated studies have shed new light that there exists distinct discontinuity and interfaces between the successive ring bands, contrary to the conventional beliefs that lamellae are continuously spiral-twist in ring-banded spherulites. Such updated views via interior lamellae dissection and analysis on banded spherulites provide avenues for circumferential cracks to develop upon cooling contraction. Spherulites are composed of complex hierarchical polycrystalline species, and they should not be viewed as being composed of straight radially-oriented lamellae or DNA-like spiraling single-crystal lamellae radiating out from a common center.

Exception of lack of correlations can be seen in circular or hexagonal circumferential cracks in some ringless spherulites. Although ring band patterns are absent (or invisible) in ringless spherulites, the cracks may be initiated near the nuclei center, where lamellae are oriented in specific patterns to guide crack to propagate. In the ringless spherulites, usually two types of circumferential cracks are common: (a) the smooth circumferential cracks; and (b) irregular and discontinuous circumferential cracks. In some rarer circumstances, spiral cracks can take a hexagon shape in the inner core, gradually transforming into circular helical in the outer periphery of spherulites. Such complex shapes of cracks are certainly hard to explain by simple directional differences in CTE, without accounting for the interior lamellae assembly that may guide the cracks. Usually, the smooth circumferential cracks are present in the ringless spherulites crystallized at lower *T*_c_ while the irregular/discontinuous cracks are in the ringless spherulites crystallized at generally higher *T*_c_. Lamellar bundles in low-*T*_c_ crystallized ringless spherulites are thinner with fewer branches, leading to cracks to propagate more smoothly. On the other hand, lamellar bundles in high-*T_c_*-crystallized ringless spherulites are much thicker with more branches and apparent twisting/scrolling, leading to cracks to propagate with intermittent interruptions. Irregular and discontinuous cracks are resulted. Cleary, the crack types, depths, and patterns in the ringless spherulites differ significantly from those in ring-banded spherulites, owing to the substantial differences of lamellae assembly in these two types of spherulites. It is critical to note that for either ring-banded or ringless spherulites of all polymers, the cracks mostly coincide with the weaker interfaces or discontinuities in the lamellae packed within the spherulites.
